# Developing and validating infant hedges for PubMed and Ovid MEDLINE: a Medical Library Association Pediatric Librarians Caucus initiative

**DOI:** 10.5195/jmla.2025.2034

**Published:** 2025-10-23

**Authors:** Lynn Kysh, Zoë Baker, Roxanne Bogucka, Emily A. Brennan, Rachel J. Hinrichs, Christine Willis

**Affiliations:** 1 lkysh@ucdavis.edu, University of California–Davis, Davis, CA; 2 zo.baker@asu.edu, Arizona State University, Tempe, AZ; 3 roxanne.bogucka@austin.utexas.edu, University of Texas—Austin, Austin, TX; 4 brennane@musc.edu, Evidence Synthesis Informationist, Medical University of South Carolina Libraries, Charleston, SC; 5 rhinrich@iu.edu, Indiana University, Indianapolis, IN; 6 christine.willis@choa.org, Children's Healthcare of Atlanta, Atlanta, GA

**Keywords:** Age Groups, Bibliographic Databases, Sensitivity and Specificity, Systematic Reviews as Topic, Validation Study

## Abstract

**Background:**

To support evidence synthesis and clinical searching, a team of librarians developed and validated infant age (birth to 23 months) search hedges for PubMed (National Library of Medicine) and Medline (OVID).

**Methods:**

We developed four sensitive hedges by selecting terms that refer to infants. Three of the hedges had identical MeSH terms and keywords but used different field tags, and the fourth was a simple keyword hedge. We compared our hedges to the built-in MeSH-based infant filter. We used relative recall calculations to validate each hedge's performance against a gold standard reference set.

**Results:**

In PubMed the similarly structured hedges performed in a range of 83.2%-83.8% sensitivity and 88.2%-89.7% specificity. The simple keyword hedge performed with an 83.5% sensitivity and 89.7% specificity. The filter generated a 70.1% sensitivity and 96.2% specificity. Similarly, in Ovid Medline, the set of similar hedges performed in a range of 82.9%-83.6% sensitivity and 88.1%-89.4% specificity. The simple keyword hedge performed with an 82.9% sensitivity and 90.8% specificity. The filter generated a 69.6% sensitivity and 96.2% specificity.

**Discussion:**

The variation in field tags did not provide a significant difference in the areas of sensitivity and specificity. The filter performed as expected with higher specificity rather than sensitivity. The simple keyword hedge performed better than anticipated with comparable sensitivity and specificity of the more complex hedges. When searching for infant population articles, the simple keyword search and filter work well for quick, clinical searching. For evidence synthesis, we recommend using one of the more sensitive infant hedges.

## INTRODUCTION

Search hedges are search strings, validated or unvalidated, on a given topic. The term “hedge“ first appeared in the literature in 1978 when Mark Funk described a “hedge” as “terms and explosions were ORed together logically, forming a… horizontally related groups of MeSH terms [1]. Many authors have commented on the nuances of terminology and definitions throughout the years. Dolan distinguished between saved searches (referred to as “saves”) and hedges. The searches that Dolan described as “saves” would now be called “filters” [2]. In 2016, Campbell proposed that the expert searcher community could use “filter” to represent the stored searches that are designed to extract articles with specific characteristics, while the term “hedge” could be used to represent stored subject searches [3]. While there is no official consensus on the definitions of these synonyms, for the purposes of this paper, “hedges” will be used as an all-encompassing term.

**Figure 1:**
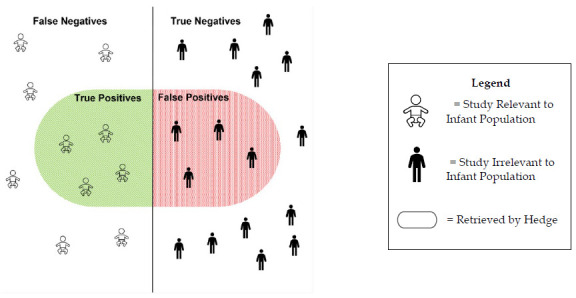
Sensitivity and Specificity of an Infant Search Hedge

Search hedges have many benefits. The first one is increased sensitivity (recall), which is the ability of the hedge to correctly identify all relevant citations about a given topic. High sensitivity means that a hedge returns most of the relevant results while irrelevant ones are also returned. Hedges increase sensitivity by expanding the search scope using synonyms, Boolean operators, truncation, wildcards, and alternate word endings and spellings. Simultaneously, hedges aim to make search queries more specific (precise) and decrease the number of irrelevant citations. High specificity means that a hedge returns more relevant results than irrelevant ones. The difference between sensitivity and specificity is visualized in Figure 1.

Librarian collaboration on systematic reviews has increased over the years [4]. By employing appropriate hedges, librarians engaging in evidence synthesis research projects can construct more effective search queries efficiently and consistently, minimizing the need for multiple search attempts. Although existing validated hedges fill an important role, sometimes the ability to revise a search hedge is also important and necessary in order to align with updates to database algorithms or to translate a validated hedge from one database to another.

In March 2021, the Medical Library Association (MLA) announced that two working groups would be “curating an open-access database of known search hedges developed by authoritative sources” and “developing a methodology for validating search hedges” [5]. The MLA Pediatric Caucus took on the latter task. The MLA Pediatric Caucus expressed a need for updated pediatric hedges because of librarians' growing collaboration on systematic reviews and the challenge of retrieving relevant pediatric literature [6–8]. Pediatric clinicians and researchers regularly encounter a paucity of relevant literature which can be traced to a lack of funding for research for children and adolescents. As early as the mid-1990s, the US congress acknowledged that inadequate resources and attention were devoted to pediatric research conducted and supported by the NIH [6]. Recent data demonstrates that the static allocation of NIH funding for pediatric research coupled with reductions in the purchasing power of budgetary funding is negatively affecting the advancement of pediatric science [6–8].

Consequently, sensitivity plays a potential role not just in systematic review methodology, but in day-to-day, or bedside, searching as well.

Our team focused on PubMed because it is a freely available and regularly used MEDLINE-based bibliographic database. PubMed also underwent a significant update in May 2020 that affected previously developed search hedges. We included Ovid Medline as it is another commonly used platform for searching MEDLINE. We opted to use the version Ovid Medline ALL for the similar comprehensiveness to PubMed in that it includes all publications from 1946 to the daily update. Although it requires a subscription, it continues to be a commonly used resource in the health sciences and provides additional features librarians rely on when conducting systematic reviews including adjacency and frequency parameters. Therefore, to create modular hedges based on age groups, the participating members from the MLA Pediatric Caucus started with an initiative to develop and validate five infant search hedges for use in PubMed and then translated them into Ovid Medline.

## MATERIALS AND METHODS

### Infant Search Hedge Development for PubMed

While we were able to locate validated hedges for the pediatric population [9–10], we were unable to locate hedges focused on the infant population. Using the infant terms located in the validated hedge, we relied on our own infant search strategies and search expertise to collaboratively develop four infant search hedges for PubMed. To create sensitive searches, we selected a wide variety of MeSH vocabulary and keywords that directly and indirectly refer to infants, including terms such as infant, neonate, neonatal, baby, newborn, infancy, preterm, premature, perinatal, postnatal, neonatology, neonatologists, NICU, and nurseries.

The search hedges ranged from what we predicted would be the most sensitive to the most specific. The first three hedges use the same subject headings (MeSH) and keywords but vary based on which keyword field tags were used. The first hedge is the most sensitive. It has no keyword field tags, which means that the keywords would be searched in many fields, and, in the case of PubMed, would be automatically mapped to additional MeSH terms and keywords. The second hedge uses text word field tags [tw], and the third hedge uses title/abstract field tags [tiab]. The fourth hedge is our “simple” search, designed to be taught to clinicians who need a quick bedside search for infants. It contains four keywords for infants with no field tags and relies on automatic term mapping for the pluralization of baby and newborn. Lastly, the fifth hedge is the database-provided infant filter for PubMed and Ovid. For reference, we present complete definitions of the utilized field tags in PubMed [11]:

**Medical Subject Headings [Mesh]:** MeSH (Medical Subject Headings) is the NLM controlled vocabulary thesaurus used for indexing PubMed citations. Use the MeSH database to find MeSH terms, including Subheadings, Publication Types, Supplementary Concepts and Pharmacological Actions, and then build a PubMed search. The MeSH database can be searched by MeSH term, MeSH Entry Term, Subheading, Publication Type, Supplementary Concept, or MeSH Scope Note.

**Title/Abstract [tiab]:** Words and numbers included in a citation's title, collection title, abstract, other abstract and author keywords (Other Term [ot] field). English language abstracts are taken directly from the published article. If an article does not have a published abstract, NLM does not create one.

**Text Words [tw]:** Includes all words and numbers in the title, abstract, other abstract, MeSH terms, MeSH Subheadings, Publication Types, Substance Names, Personal Name as Subject, Corporate Author, Secondary Source, Comment/Correction Notes, and Other Terms typically non-MeSH subject terms (keywords)

### Infant Search Hedge Development for Ovid Medline All

After completing the development and validation of the PubMed infant search hedges, we translated them for the Ovid Medline interface and referred to Ovid's MEDLINE database guide for field tag definitions and functions [12]. To preserve the first hedge as the most sensitive, we opted to use the .af or all fields searchable fields tag. For the second hedge, we translated the PubMed (tiab) field tag to the default .mp or multi-purpose field tag which includes abstract, anatomy supplementary concept, book title, floating sub-heading word, keyword heading word, name of substance word, organism supplementary concept word, original title, population supplementary concept word, protocol supplementary concept word, rare disease supplementary concept word, subject heading word, synonyms, title, and unique identifier. For the third hedge, we translated the PubMed text word (tw) to the ab,kf,ti field tags trio which includes the indexed abstract, author-supplied keywords, and title words. With the goal of sensitivity in mind, we chose the keyword heading word-indexed field tag (.kf) rather than keyword heading phrase-indexed field tag (.kw) because .kf allows us to capture any instance of our search terms rather than an exact phrase. We opted for title (.ti) rather than original title (.ot) given that our team's language proficiency is limited to English. To maintain the “simple” nature of the fourth hedge, we chose the multi-purpose field tag as it is the default setting in a basic Ovid search and included babies and newborns as they would not be automatically included as they were in PubMed with automatic term mapping. And lastly, we used the Ovid age group limit for all infants from birth to 23 months which is the equivalent to the PubMed infant age filter. See Table 1 for all infant search hedges for PubMed (see Appendix A for all infant search hedges for Ovid Medline).

**Table 1:** Infant Search Hedges for PubMed

**Search Hedge #1:** (“Infant”[Mesh] OR “Infant Health”[Mesh] OR “Infant Welfare”[Mesh] OR “Infant Death”[Mesh] OR “Sudden Infant Death”[Mesh] OR “Infant Mortality”[Mesh] OR “Infant Behavior”[Mesh] OR “Infant Care”[Mesh] OR “Infant, Newborn”[Mesh] OR “Infant, Low Birth Weight”[Mesh] OR “Infant, Small for Gestational Age”[Mesh] OR “Infant, Very Low Birth Weight”[Mesh] OR “Infant, Extremely Low Birth Weight”[Mesh] OR infant OR infants OR infantile OR infancy OR infantile OR “Infant, Postmature”[Mesh] OR “Infant, Premature”[Mesh] OR “Infant, Extremely Premature”[Mesh] OR “Premature Birth”[Mesh] OR premature OR prematurity OR preterm OR pre-term OR premie OR premies OR perinatal OR peri-natal OR perinat* OR “Perinatal Death”[Mesh] OR “Perinatal Mortality”[Mesh] OR “Perinatal Care”[Mesh] OR “Postnatal Care”[Mesh] OR postnatal OR post-natal OR postnatal* OR newborn OR newborns OR neonate OR neonates OR neonatal OR neonatale OR neonatales OR neonatle OR neonatles OR neonatally OR neonatorum OR “Neonatal Screening”[Mesh] OR “Neonatology”[Mesh] OR “Neonatologists”[Mesh] OR “Neonatal Nursing”[Mesh] OR “Nurses, Neonatal”[Mesh] OR neonatology OR neonatologist OR neonatologists OR “Intensive Care, Neonatal”[Mesh] OR “Intensive Care Units, Neonatal”[Mesh] OR NICU OR NICUs OR “Neonatal Screening”[Mesh] OR “Nurseries, Infant”[Mesh] OR “Nurseries, Hospital”[Mesh] OR nursery OR nurseries OR baby OR babies)
**Search Hedge #2:** (“Infant”[Mesh] OR “Infant Health”[Mesh] OR “Infant Welfare”[Mesh] OR “Infant Death”[Mesh] OR “Sudden Infant Death”[Mesh] OR “Infant Mortality”[Mesh] OR “Infant Behavior”[Mesh] OR “Infant Care”[Mesh] OR “Infant, Newborn”[Mesh] OR “Infant, Low Birth Weight”[Mesh] OR “Infant, Small for Gestational Age”[Mesh] OR “Infant, Very Low Birth Weight”[Mesh] OR “Infant, Extremely Low Birth Weight”[Mesh] OR infant[tw] OR infants[tw] OR infantile[tw] OR infancy[tw] OR infantile[tw] OR “Infant, Postmature”[Mesh] OR “Infant, Premature”[Mesh] OR “Infant, Extremely Premature”[Mesh] OR “Premature Birth”[Mesh] OR premature[tw] OR prematurity[tw] OR preterm[tw] OR pre-term[tw] OR premie[tw] OR premies[tw] OR perinatal[tw] OR peri-natal[tw] OR perinat*[tw] OR “Perinatal Death”[Mesh] OR “Perinatal Mortality”[Mesh] OR “Perinatal Care”[Mesh] OR “Postnatal Care”[Mesh] OR postnatal[tw] OR post-natal[tw] OR postnatal*[tw] OR newborn[tw] OR newborns[tw] OR neonate[tw] OR neonates[tw] OR neonatal[tw] OR neonatale[tw] OR neonatales[tw] OR neonatle[tw] OR neonatles[tw] OR neonatally[tw] OR neonatorum[tw] OR “Neonatal Screening”[Mesh] OR “Neonatology”[Mesh] OR “Neonatologists”[Mesh] OR “Neonatal Nursing”[Mesh] OR “Nurses, Neonatal”[Mesh] OR neonatology[tw] OR neonatologist[tw] OR neonatologists[tw] OR “Intensive Care, Neonatal”[Mesh] OR “Intensive Care Units, Neonatal”[Mesh] OR NICU[tw] OR NICUs[tw] OR “Neonatal Screening”[Mesh] OR “Nurseries, Infant”[Mesh] OR “Nurseries, Hospital”[Mesh] OR nursery[tw] OR nurseries[tw] OR baby[tw] OR babies[tw]
**Search Hedge #3:** (“Infant”[Mesh] OR “Infant Health”[Mesh] OR “Infant Welfare”[Mesh] OR “Infant Death”[Mesh] OR “Sudden Infant Death”[Mesh] OR “Infant Mortality”[Mesh] OR “Infant Behavior”[Mesh] OR “Infant Care”[Mesh] OR “Infant, Newborn”[Mesh] OR “Infant, Low Birth Weight”[Mesh] OR “Infant, Small for Gestational Age”[Mesh] OR “Infant, Very Low Birth Weight”[Mesh] OR “Infant, Extremely Low Birth Weight”[Mesh] OR infant[tiab] OR infants[tiab] OR infantile[tiab] OR infancy[tiab] OR infantile[tiab] OR “Infant, Postmature”[Mesh] OR “Infant, Premature”[Mesh] OR “Infant, Extremely Premature”[Mesh] OR “Premature Birth”[Mesh] OR premature[tiab] OR prematurity[tiab] OR preterm[tiab] OR pre-term[tiab] OR premie[tiab] OR premies[tiab] OR perinatal[tiab] OR peri-natal[tiab] OR perinat*[tiab] OR “Perinatal Death”[Mesh] OR “Perinatal Mortality”[Mesh] OR “Perinatal Care”[Mesh] OR “Postnatal Care”[Mesh] OR postnatal[tiab] OR post-natal[tiab] OR postnatal*[tiab] OR newborn[tiab] OR newborns[tiab] OR neonate[tiab] OR neonates[tiab] OR neonatal[tiab] OR neonatale[tiab] OR neonatales[tiab] OR neonatle[tiab] OR neonatles[tiab] OR neonatally[tiab] OR neonatorum[tiab] OR “Neonatal Screening”[Mesh] OR “Neonatology”[Mesh] OR “Neonatologists”[Mesh] OR “Neonatal Nursing”[Mesh] OR “Nurses, Neonatal”[Mesh] OR neonatology[tiab] OR neonatologist[tiab] OR neonatologists[tiab] OR “Intensive Care, Neonatal”[Mesh] OR “Intensive Care Units, Neonatal”[Mesh] OR NICU[tiab] OR NICUs[tiab] OR “Neonatal Screening”[Mesh] OR “Nurseries, Infant”[Mesh] OR “Nurseries, Hospital”[Mesh] OR nursery[tiab] OR nurseries[tiab] OR baby[tiab] OR babies[tiab])
**Search Hedge #4:** (infan* OR baby OR neonat* OR newborn)
**Search Hedge #5:** Infant[MeSH]

### Developing a Gold Standard Reference Set

Next, we developed a gold standard reference set of articles to test our infant hedges. A gold standard reference set would include only articles with a true infant population (birth to 23 months). To create this set, we identified five search topics that retrieve references on adult and infant populations: pulmonary hypertension, hypoglycemia, cerebral palsy, sepsis, and brain hypoxia-ischemia. The search strategies for these topics used a combination of MeSH and keywords (see Appendix A). No filters were applied but we did include the Cochrane human study hedge to decrease the number of animal studies retrieved [13]. We initially gathered 200 references on each of the five topics, giving us 1000 total. We chose to include a mix of older and newly published articles to capture articles that have been indexed and those that have not yet been indexed to simulate typical searching landscape. Although the National Library of Medicine announced the transition to automated MeSH indexing, [12] there continues to be a greater than 24-hour lag time between when citations are added to PubMed and when those citations are fully indexed to include MeSH terms to increase their discoverability. For each topic, we ran a PubMed search on June 15, 2021, sorted results by publication date and exported the 100 most recent references. Next, we applied the custom date range of 1/1/16-12/31/16, sorted by publication date, and exported the first 100 references for each of the five topics. References were exported and archived in the citation management software EndNote 20 (Clarivate Analytics, Philadelphia, PA, United States) and then imported into the review management software, Covidence (Covidence systematic review software, Veritas Health Innovation, Melbourne, Australia).

To test the screening process and determine inter-rater reliability, we performed a pilot test on 50 of the 1000 references. In Covidence, we sorted the 1000 references by author's last name (A-Z) and exported the first 50 to Excel. Five reviewers (EB, RB, RH, LK, CW) independently screened each reference and indicated (yes, no, unsure) if the reference included a human infant population aged birth-23 month. All the reviewers met to discuss discrepancies and further refine the eligibility criteria for what a “true positive” infant article was. We determined that articles would be included if they had an infant population (birth-23 months) or if an infant population was included in the study's inclusion criteria, but the sample did not include infants. We also included articles on family-centered care involving infants, and articles with pediatric or adult outcomes related to neonatal diagnoses. Included articles could be any type of publication or study, including corrections, editorials, and commentaries. We determined that articles would be excluded if they included a population older than 24 months, were bench research articles, were not in English, were animal studies, or discussed maternal outcomes only. Following these criteria, fifteen of the 50 references from the test set were identified as “true positive” infant articles [14].

The reviewers then screened the total set of 1000 references in Covidence. The same five reviewers who conducted the pilot project screening conducted this project screening. The title and abstract of each reference were independently screened by two reviewers (any two). Conflicts were resolved by a third reviewer (not a screener). Following the same process, reviewers then independently screened full-text articles with conflicts being resolved by a third reviewer. After completing a preliminary analysis of the PubMed search hedges, the team reconvened in April 2023 to complete the Ovid Medline analysis. Given the time that had passed, the collection of recently published citations had been indexed to include MeSH terms and the team realized the gold standard had lost its simulation of the everyday searching landscape. To correct this expiration of most recently published studies, on April 7, 2023, we conducted a new search in PubMed on the same five pre-identified topics with the same search strategies and sorted results by publication date and exported the 100 most recent references. The same team of reviewers completed the title and abstract screening process and full-text screening process in Covidence creating a new total set of 1500 references. After screening, we determined that 291 articles included an infant population and 1,209 did not. The set of 291 articles is our true positive gold standard reference set [14].

### Gold Standard Search Hedge Analysis

We generated true positive and false positive values by running each search hedge in both PubMed and Ovid Medline. For true positives, we ran each search hedge in the database and combined it with the collection of pre-identified positive PMIDs using the Boolean operator “and”. The resulting number of search results was the number of true positives generated from the hedge (Hedge AND +PMIDs). We then calculated the false negative value by subtracting the number of true positives from the number of pre-identified positive PMIDs. For false positives, we ran each search hedge in the database and combined it with the collection of pre-identified negative PMIDs using the Boolean operator “and”. The resulting number of search results was the number of false positives generated from the hedge. We then calculated the true negative value by subtracting the number of false positives from the number of pre-identified negative PMIDs. See Table 2 for a summary of equations used [15].

**Table 2:** Summary of Equations for Gold Standard Search Hedge Analysis

**True Positive** *Hedge AND + PMIDs*	**False Positive** *Hedge AND − PMIDs*
**False Negative** *# of + PMIDs − (Hedge AND + PMIDs)*	**True Negative** *# of − PMIDs − (Hedge AND − PMIDs)*

### Data Analysis

The pilot project produced an inter-rater reliability of .54. However, we learned that four of the group members consulted full-text if needed, and one reviewer did not. This was an issue of miscommunication, and we resolved it so that all group members consulted full text if needed. If we remove that one reviewer from the equation, the inter-rater reliability changes to 0.8.

Specificity, sensitivity, positive predictive value (PPV), negative predictive value (NPV), and overall accuracy were calculated for all five hedges for both PubMed and Ovid Medline utilizing standard formulas. True positive findings were defined as references identified by the hedge as including a human infant population that truly did include a human infant population upon reviewer screening. True negative findings were defined as references not identified by the hedge as including a human infant population that truly did not include a human infant population upon screening. False positive findings were defined as references identified by the hedge as including a human infant population that did not include a human infant population upon screening. False negative findings were defined as references not identified by the hedge as including a human infant population that did include a human infant population upon reviewer screening.

As such, sensitivity reflects the hedge correctly identifying articles that include human infants, while specificity reflects the hedge correctly excluding articles that do not include human infants. Similarly, PPV reflects the proportion of articles identified by the hedge as including infants that truly included infants, while NPV reflects the proportion of the articles excluded by the hedge that truly did not include infants. Overall accuracy was defined as the proportion of correctly identified (true positive and true negative) articles, out of all screened articles.

When analyzing PubMed findings, calculations were performed utilizing 1,500 unique references, with all articles in PubMed assigned a unique PMID to automatically exclude duplications. As Ovid Medline may not exclude duplications of non-indexed citations through a unique identifier in the case of pre-publications being available concurrently with final versions of the same manuscript, duplications may be present. As this reflects a real-world scenario, the decision was made to analyze Ovid Medline findings using the denominator of 1,506 references, without manual exclusion of 6 duplicates. Hedges' sensitivity and specificity in identifying articles including infants utilizing PubMed and Ovid Medline searches were displayed graphically.

## RESULTS

In PubMed, the search strategies generated sensitivity levels between 83.2%-83.8% and specificity between 88.2%-89.7%. The exception was the built in PubMed filter which generated 70.1% sensitivity and 96.2% specificity (see Table 3). In OVID Medline, the search strategies generated sensitivity levels between 82.9%-83.6% and specificity levels between 88.1%-89.4%. The exception was the built-in filter which generated 69.6% sensitivity and 96.2% specificity (see Appendix A). See Table 3.

**Table 3:** Sensitivity and Specificity in PubMed

	Sensitivity	Specificity	Positive Predictive Value (PPV)	Negative Predictive Value (NPV)
Hedge 1: No Keyword Field Tags	83.8%	88.2%	63.0%	95.8%
Hedge 2: Text Word Field Tags	83.2%	89.3%	65.2%	95.7%
Hedge 3: Title/Abstract Field Tags	83.2%	89.4%	65.4%	95.7%
Hedge 4: Simple	83.5%	89.7%	66.0%	95.8%
Hedge 5: PubMed Infant Filter	70.1%	96.2%	81.6%	93.0%

## DISCUSSION

Hedge 1 (no keyword field tags) had the highest sensitivity (83.8% in PubMed and 83.6% in Ovid Medline) and therefore it may be best for use in systematic and scoping reviews. However, Hedges 1 through 4 had similar sensitivity. Because these data do not suggest any clear advantages we encourage librarians to explore each of these hedges by testing them with other search concepts and reflect on the role of sensitivity and specificity for their specific information needs. For example, Hedge 5 (the PubMed infant filter) is the most specific, so is most appropriate for bedside searching. Meanwhile, Hedge 4 (the simple search) has high sensitivity and specificity, offering a balance between recall and precision, which makes it applicable for both bedside searches and potentially classroom instruction.

Our reported values for sensitivity and specificity may appear low relative to other hedge validation studies, [16,17]. This may be due to the subject of pediatrics. Studies limited to the adult population do not typically describe their inclusion criteria by age as transparently as pediatric studies, which can have an impact on the overall precision of a search strategy, especially when the indexing with a controlled vocabulary is pending for a citation. An infant hedge poses an additional and similar challenge in that studies are more likely to include caregiver or maternal outcomes rather than infant outcomes, which would not meet the inclusion criteria of our proposed hedges.

The primary intention of this project was to create a transparent search hedge that can be used for systematic or scoping review studies as well as benefit bedside search requests. We selected the subject areas to test based on overlap with a variety of ages in order to confirm both the sensitivity and specificity of the selected infant terms. Testing these search hedges in both PubMed and Ovid Medline databases confirmed that the terms are transferrable with the appropriate search tags applied. It should be noted that the PubMed infant filter works best with articles that are already indexed as it relies solely on the automated explosion of Infant[Mesh].

**Figure 2:**
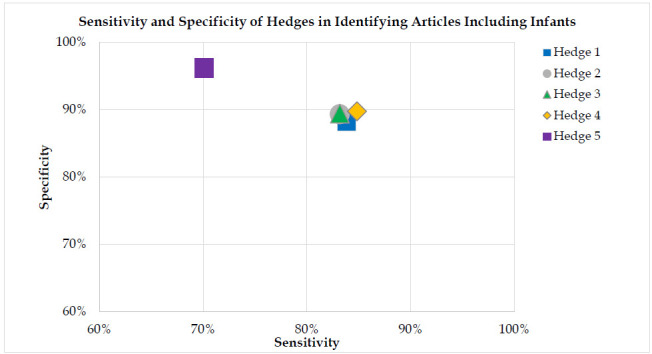
Sensitivity and Specificity of Hedges in Identifying Articles Including Infants in PubMed

**Figure 3:**
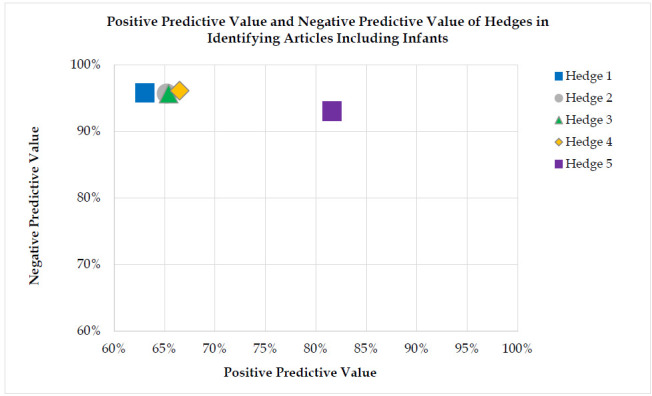
Positive Predictive Value and Negative Predictive Value of Hedges in Identifying Articles Including Infants in PubMed

## LIMITATIONS

This study has several limitations. These infant hedges were validated in PubMed and Ovid Medline but may not be generalizable to other databases. PubMed utilizes automatic term mapping, which is why the simple search hedge (#4) was so effective [18]. We suspect that this would not be the case in databases with less sophisticated search algorithms. Also, these search hedges were tested and validated in the current version of PubMed, so any changes in PubMed's search algorithm could impact these research findings. The performance of these search hedges could differ between clinical topics versus social topics. Clinical studies are likely to refer to infant populations using keywords such as infant or newborn or neonate. However, studies that are more social sciences leaning, may not refer to the infant population by name. For example, articles about early intervention strategies may not specifically refer to infant populations, although they would be relevant to that population.

Lastly, although the team shares a combined experience of decades of experience in interprofessional collaboration in supporting pediatric clinicians and researchers we acknowledge that this is not equivalent to the formalized education and training necessary to determine every potential clinician's information needs. Consequently, the lack of this described clinical subject expertise by librarian screeners could have affected decision making while screening. For example, sometimes we were unclear if an article was truly basic science research or not, or if a study's inclusion criteria fit our infant age range, but the study itself did not actually enroll patients in our specified age range, there may have been disagreements about whether that particular study should be included or not. Future projects could potentially benefit from the participation of pediatricians and/or pediatric researchers, especially in regard to the screening process.

## CONCLUSIONS

This project focused on the development and validation of sensitive infant search hedges for use in PubMed and Ovid Medline. By providing a transparent and reproducible validation process, this project serves as a valuable framework for others interested in developing and validating search hedges. The methodological details and supporting materials, such as an Excel template for calculating sensitivity and specificity, are housed in Open Science Framework [14]. We recommend that teams taking on a similar project benefit from our lessons learned which include ensuring that you have the appropriate resources of interprofessional expertise and appropriate software including a shared citation management software. Teams interested in conducting a comparison between multiple databases should conduct test searches on the same date to ensure comparability as databases are not stagnant and undergo additions and developments.

The MLA Pediatric Caucus will continue to use this methodology to develop and validate hedges for other pediatric age subsets for PubMed and Ovid Medline. The MLA Pediatric Caucus's larger goal is the development of modular age-based search hedges which will allow librarians and researchers to combine hedges or use them individually. The MLA Pediatric Caucus will also be accountable to maintaining the validity of the developed search hedges by responding to likely algorithmic changes in PubMed and Ovid Medline as well as the less likely social and linguistical changes in how pediatric populations are described.

## Data Availability

Search hedges and citation collections are available under controlled access through the Open Science Framework (OSF): https://osf.io/hbvjm/ under Creative Commons Attribution-Share Alike (CC BY-SA).
