# Supplementary material for: Developing and validating infant hedges for PubMed and Ovid MEDLINE: a Medical Library Association Pediatric Librarians Caucus initiative
Source: J Med Libr Assoc. 2025 Oct 23;113(4):281–9. doi: 10.5195/jmla.2025.2034 (PMC12604066; doi:10.5195/jmla.2025.2034)
Supplement: Supplementary file 1 — Appendix A [file jmla-113-4-281-s01.docx]

**Infant Search Hedges for PubMed**

| **Search Hedge #1:** ("Infant"[Mesh] OR "Infant Health"[Mesh] OR "Infant Welfare"[Mesh] OR "Infant Death"[Mesh] OR "Sudden Infant Death"[Mesh] OR "Infant Mortality"[Mesh] OR "Infant Behavior"[Mesh] OR "Infant Care"[Mesh] OR "Infant, Newborn"[Mesh] OR "Infant, Low Birth Weight"[Mesh] OR "Infant, Small for Gestational Age"[Mesh] OR "Infant, Very Low Birth Weight"[Mesh] OR "Infant, Extremely Low Birth Weight"[Mesh] OR infant OR infants OR infantile OR infancy OR infantile OR "Infant, Postmature"[Mesh] OR "Infant, Premature"[Mesh] OR "Infant, Extremely Premature"[Mesh] OR "Premature Birth"[Mesh] OR premature OR prematurity OR preterm OR pre-term OR premie OR premies OR perinatal OR peri-natal OR perinat* OR "Perinatal Death"[Mesh] OR "Perinatal Mortality"[Mesh] OR "Perinatal Care"[Mesh] OR "Postnatal Care"[Mesh] OR postnatal OR post-natal OR postnatal* OR newborn OR newborns OR neonate OR neonates OR neonatal OR neonatale OR neonatales OR neonatle OR neonatles OR neonatally OR neonatorum OR "Neonatal Screening"[Mesh] OR "Neonatology"[Mesh] OR "Neonatologists"[Mesh] OR "Neonatal Nursing"[Mesh] OR "Nurses, Neonatal"[Mesh] OR neonatology OR neonatologist OR neonatologists OR "Intensive Care, Neonatal"[Mesh] OR "Intensive Care Units, Neonatal"[Mesh] OR NICU OR NICUs OR "Neonatal Screening"[Mesh] OR "Nurseries, Infant"[Mesh] OR "Nurseries, Hospital"[Mesh] OR nursery OR nurseries OR baby OR babies) |
| --- |
| **Search Hedge #2:** ("Infant"[Mesh] OR "Infant Health"[Mesh] OR "Infant Welfare"[Mesh] OR "Infant Death"[Mesh] OR "Sudden Infant Death"[Mesh] OR "Infant Mortality"[Mesh] OR "Infant Behavior"[Mesh] OR "Infant Care"[Mesh] OR "Infant, Newborn"[Mesh] OR "Infant, Low Birth Weight"[Mesh] OR "Infant, Small for Gestational Age"[Mesh] OR "Infant, Very Low Birth Weight"[Mesh] OR "Infant, Extremely Low Birth Weight"[Mesh] OR infant[tw] OR infants[tw] OR infantile[tw] OR infancy[tw] OR infantile[tw] OR "Infant, Postmature"[Mesh] OR "Infant, Premature"[Mesh] OR "Infant, Extremely Premature"[Mesh] OR "Premature Birth"[Mesh] OR premature[tw] OR prematurity[tw] OR preterm[tw] OR pre-term[tw] OR premie[tw] OR premies[tw] OR perinatal[tw] OR peri-natal[tw] OR perinat*[tw] OR "Perinatal Death"[Mesh] OR "Perinatal Mortality"[Mesh] OR "Perinatal Care"[Mesh] OR "Postnatal Care"[Mesh] OR postnatal[tw] OR post-natal[tw] OR postnatal*[tw] OR newborn[tw] OR newborns[tw] OR neonate[tw] OR neonates[tw] OR neonatal[tw] OR neonatale[tw] OR neonatales[tw] OR neonatle[tw] OR neonatles[tw] OR neonatally[tw] OR neonatorum[tw] OR "Neonatal Screening"[Mesh] OR "Neonatology"[Mesh] OR "Neonatologists"[Mesh] OR "Neonatal Nursing"[Mesh] OR "Nurses, Neonatal"[Mesh] OR neonatology[tw] OR neonatologist[tw] OR neonatologists[tw] OR "Intensive Care, Neonatal"[Mesh] OR "Intensive Care Units, Neonatal"[Mesh] OR NICU[tw] OR NICUs[tw] OR "Neonatal Screening"[Mesh] OR "Nurseries, Infant"[Mesh] OR "Nurseries, Hospital"[Mesh] OR nursery[tw] OR nurseries[tw] OR baby[tw] OR babies[tw] |
| **Search Hedge #3:** ("Infant"[Mesh] OR "Infant Health"[Mesh] OR "Infant Welfare"[Mesh] OR "Infant Death"[Mesh] OR "Sudden Infant Death"[Mesh] OR "Infant Mortality"[Mesh] OR "Infant Behavior"[Mesh] OR "Infant Care"[Mesh] OR "Infant, Newborn"[Mesh] OR "Infant, Low Birth Weight"[Mesh] OR "Infant, Small for Gestational Age"[Mesh] OR "Infant, Very Low Birth Weight"[Mesh] OR "Infant, Extremely Low Birth Weight"[Mesh] OR infant[tiab] OR infants[tiab] OR infantile[tiab] OR infancy[tiab] OR infantile[tiab] OR "Infant, Postmature"[Mesh] OR "Infant, Premature"[Mesh] OR "Infant, Extremely Premature"[Mesh] OR "Premature Birth"[Mesh] OR premature[tiab] OR prematurity[tiab] OR preterm[tiab] OR pre-term[tiab] OR premie[tiab] OR premies[tiab] OR perinatal[tiab] OR peri-natal[tiab] OR perinat*[tiab] OR "Perinatal Death"[Mesh] OR "Perinatal Mortality"[Mesh] OR "Perinatal Care"[Mesh] OR "Postnatal Care"[Mesh] OR postnatal[tiab] OR post-natal[tiab] OR postnatal*[tiab] OR newborn[tiab] OR newborns[tiab] OR neonate[tiab] OR neonates[tiab] OR neonatal[tiab] OR neonatale[tiab] OR neonatales[tiab] OR neonatle[tiab] OR neonatles[tiab] OR neonatally[tiab] OR neonatorum[tiab] OR "Neonatal Screening"[Mesh] OR "Neonatology"[Mesh] OR "Neonatologists"[Mesh] OR "Neonatal Nursing"[Mesh] OR "Nurses, Neonatal"[Mesh] OR neonatology[tiab] OR neonatologist[tiab] OR neonatologists[tiab] OR "Intensive Care, Neonatal"[Mesh] OR "Intensive Care Units, Neonatal"[Mesh] OR NICU[tiab] OR NICUs[tiab] OR "Neonatal Screening"[Mesh] OR "Nurseries, Infant"[Mesh] OR "Nurseries, Hospital"[Mesh] OR nursery[tiab] OR nurseries[tiab] OR baby[tiab] OR babies[tiab]) |
| **Search Hedge #4:** (infan* OR baby OR neonat* OR newborn) |
| **Search Hedge #5:** Infant[MeSH] |

**Infant Search Hedges for Ovid Medline**

| **Search Hedge #1:** (Infant/ OR Infant Health/ OR Infant Welfare/ OR Infant Death/ OR Sudden Infant Death/ OR Infant Mortality/ OR Infant Behavior/ OR Infant Care/ OR Infant, Newborn/ OR Infant, Low Birth Weight/ OR Infant, Small for Gestational Age/ OR Infant, Very Low Birth Weight/ OR Infant, Extremely Low Birth Weight/ OR infant.af OR infants.af OR infantile.af OR infancy.af OR "Infant, Postmature"/ OR "Infant, Premature"/ OR "Infant, Extremely Premature"/ OR "Premature Birth"/ OR premature.af OR prematurity.af OR preterm.af OR pre-term.af OR premie.af OR premies.af OR perinatal.af OR peri-natal.af OR perinat*.af OR "Perinatal Death"/ OR "Perinatal Mortality"/ OR "Perinatal Care"/ OR "Postnatal Care"/ OR postnatal.af OR post-natal.af OR postnatal*.af OR newborn.af OR newborns.af OR neonate.af OR neonates.af OR neonatal.af OR neonatale.af OR neonatales.af OR neonatle.af OR neonatles.af OR neonatally.af OR neonatorum.af OR Neonatal Screening/ OR Neonatology/ OR Neonatologists/ OR Neonatal Nursing/ OR Nurses, Neonatal/ OR neonatology.af OR neonatologist.af OR neonatologists.af OR Intensive Care, Neonatal/ OR Intensive Care Units, Neonatal/ OR NICU.af OR NICUs.af OR Neonatal Screening/ OR Nurseries, Infant/ OR Nurseries, Hospital/ OR nursery.af OR nurseries.af OR baby.af OR babies.af) |
| --- |
| **Search Hedge #2:** (Infant/ OR Infant Health/ OR Infant Welfare/ OR Infant Death/ OR Sudden Infant Death/ OR Infant Mortality/ OR Infant Behavior/ OR Infant Care/ OR Infant, Newborn/ OR Infant, Low Birth Weight/ OR Infant, Small for Gestational Age/ OR Infant, Very Low Birth Weight/ OR Infant, Extremely Low Birth Weight/ OR infant.mp OR infants.mp OR infantile.mp OR infancy.mp OR "Infant, Postmature"/ OR "Infant, Premature"/ OR "Infant, Extremely Premature"/ OR "Premature Birth"/ OR premature.mp OR prematurity.mp OR preterm.mp OR pre-term.mp OR premie.mp OR premies.mp OR perinatal.mp OR peri-natal.mp OR perinat*.mp OR "Perinatal Death"/ OR "Perinatal Mortality"/ OR "Perinatal Care"/ OR "Postnatal Care"/ OR postnatal.mp OR post-natal.mp OR postnatal*.mp OR newborn.mp OR newborns.mp OR neonate.mp OR neonates.mp OR neonatal.mp OR neonatale.mp OR neonatales.mp OR neonatle.mp OR neonatles.mp OR neonatally.mp OR neonatorum.mp OR Neonatal Screening/ OR Neonatology/ OR Neonatologists/ OR Neonatal Nursing/ OR Nurses, Neonatal/ OR neonatology.mp OR neonatologist.mp OR neonatologists.mp OR Intensive Care, Neonatal/ OR Intensive Care Units, Neonatal/ OR NICU.mp OR NICUs.mp OR Neonatal Screening/ OR Nurseries, Infant/ OR Nurseries, Hospital/ OR nursery.mp OR nurseries.mp OR baby.mp OR babies.mp) |
| **Search Hedge #3:**  (Infant/ OR Infant Health/ OR Infant Welfare/ OR Infant Death/ OR Sudden Infant Death/ OR Infant Mortality/ OR Infant Behavior/ OR Infant Care/ OR Infant, Newborn/ OR Infant, Low Birth Weight/ OR Infant, Small for Gestational Age/ OR Infant, Very Low Birth Weight/ OR Infant, Extremely Low Birth Weight/ OR infant.ab,kf,ti OR infants.ab,kf,ti OR infantile.ab,kf,ti OR infancy.ab,kf,ti OR "Infant, Postmature"/ OR "Infant, Premature"/ OR "Infant, Extremely Premature"/ OR "Premature Birth"/ OR premature.ab,kf,ti OR prematurity.ab,kf,ti OR preterm.ab,kf,ti OR pre-term.ab,kf,ti OR premie.ab,kf,ti OR premies.ab,kf,ti OR perinatal.ab,kf,ti OR peri-natal.ab,kf,ti OR perinat*.ab,kf,ti OR "Perinatal Death"/ OR "Perinatal Mortality"/ OR "Perinatal Care"/ OR "Postnatal Care"/ OR postnatal.ab,kf,ti OR post-natal.ab,kf,ti OR postnatal*.ab,kf,ti OR newborn.ab,kf,ti OR newborns.ab,kf,ti OR neonate.ab,kf,ti OR neonates.ab,kf,ti OR neonatal.ab,kf,ti OR neonatale.ab,kf,ti OR neonatales.ab,kf,ti OR neonatle.ab,kf,ti OR neonatles.ab,kf,ti OR neonatally.ab,kf,ti OR neonatorum.ab,kf,ti OR Neonatal Screening/ OR Neonatology/ OR Neonatologists/ OR Neonatal Nursing/ OR Nurses, Neonatal/ OR neonatology.ab,kf,ti OR neonatologist.ab,kf,ti OR neonatologists.ab,kf,ti OR Intensive Care, Neonatal/ OR Intensive Care Units, Neonatal/ OR NICU.ab,kf,ti OR NICUs.ab,kf,ti OR Neonatal Screening/ OR Nurseries, Infant/ OR Nurseries, Hospital/ OR nursery.ab,kf,ti OR nurseries.ab,kf,ti OR baby.ab,kf,ti OR babies.ab,kf,ti) |
| **Search Hedge #4:** (infan* or baby or babies or neonat* or newborn or newborns).mp. |
| **Search Hedge #5:** limit to “all infant (birth to 23 months)” |

**Sensitivity and Specificity in Ovid Medline**

|  | Sensitivity | Specificity | Positive Predictive Value (PPV) | Negative Predictive Value (NPV) |
| --- | --- | --- | --- | --- |
| Hedge 1: All Fields Tags | 83.6% | 88.1% | 63.0% | 95.7% |
| Hedge 2: Multipurpose Field Tags | 82.9% | 89.4% | 65.3% | 95.6% |
| Hedge 3: Abstract, Keyword Heading, Title Field Tags | 82.9% | 89.3% | 65.1% | 95.6% |
| Hedge 4: Simple | 82.9% | 90.8% | 68.5% | 95.7% |
| Hedge 5: Ovid Medline Infant Filter | 69.6% | 96.2% | 81.6% | 92.9% |

**Searches to Develop Gold Standard Reference Set:**

- ("Hypertension, Pulmonary"[Mesh] OR "pulmonary hypertension") NOT ("Animals"[Mesh] NOT "Humans"[Mesh]) Sort by: Publication Date
- ("Hypertension, Pulmonary"[Mesh] OR "pulmonary hypertension") NOT ("Animals"[Mesh] NOT "Humans"[Mesh]) Filters: from 2016/1/1 - 2016/12/31 Sort by: Publication Date
- ("Hypoglycemia"[Mesh] OR hypoglycemia) NOT ("Animals"[Mesh] NOT "Humans"[Mesh]) Sort by: Publication Date
- ("Hypoglycemia"[Mesh] OR hypoglycemia) NOT ("Animals"[Mesh] NOT "Humans"[Mesh]) Filters: from 2016/1/1 - 2016/12/31 Sort by: Publication Date
- ("Cerebral Palsy"[Mesh] OR cerebral palsy) NOT ("Animals"[Mesh] NOT "Humans"[Mesh]) Filters: from 2016/1/1 - 2016/12/31 Sort by: Publication Date
- ("Cerebral Palsy"[Mesh] OR cerebral palsy) NOT ("Animals"[Mesh] NOT "Humans"[Mesh]) Filters: from 2016/1/1 - 2016/12/31 Sort by: Publication Date
- ("Sepsis"[Mesh:NoExp] OR "Neonatal Sepsis"[Mesh] OR sepsis) NOT ("Animals"[Mesh] NOT "Humans"[Mesh]) Sort by: Publication Date
- ("Sepsis"[Mesh:NoExp] OR "Neonatal Sepsis"[Mesh] OR sepsis) NOT ("Animals"[Mesh] NOT "Humans"[Mesh]) Filters: from 2016/1/1 - 2016/12/31 Sort by: Publication Date
- ("Hypoxia-Ischemia, Brain"[Mesh] OR brain hypoxia-ischemia) NOT ("Animals"[Mesh] NOT "Humans"[Mesh]) Sort by: Publication Date
- ("Hypoxia-Ischemia, Brain"[Mesh] OR brain hypoxia-ischemia) NOT ("Animals"[Mesh] NOT "Humans"[Mesh]) Filters: from 2016/1/1 - 2016/12/31 Sort by: Publication Date

**Sensitivity and Specificity of Hedges in Identifying Articles Including Infants in Ovid Medline**

**Positive Predictive Value and Negative Predictive Value of Hedges in Identifying Articles Including Infants in Ovid Medline**
